# Ultrasound-Guided Genicular Nerve Block: Novel Clinical Role for Postoperative Knee Replacement Surgery Pain Management

**DOI:** 10.1007/s11916-026-01548-7

**Published:** 2026-08-20

**Authors:** David G. Cooper, Charles P. Daniel, Alan D. Kaye

**Affiliations:** 1 Department of Anesthesiology, Tulane University School of Medicine, 1430 Tulane Avenue, New Orleans, LA USA; 2 Department of Anesthesiology, Louisiana State University School of Medicine, 1501 Kings Highway, Shreveport, LA 71103 USA; 3 Departments of Anesthesiology and Pharmacology, Toxicology, and Neurosciences, Louisiana State University Health Sciences Center Shreveport, Shreveport, LA 71103 USA

**Keywords:** Ultrasound, Analgesia, Genicular, Knee, Post-surgical pain

## Abstract

**Purpose of Review:**

Important aspects of knee replacement surgery to consider are regional anesthesia techniques and postoperative analgesia and ambulation.

**Recent Findings:**

To maximize targeted pain relief for knee replacement surgery while minimizing postoperative opioid consumption, ultrasound-guided regional anesthesia nerve blocks have been utilized over the past decade. Recent clinical studies have demonstrated efficacy with minimal risks. The genicular nerve block has emerged recently as a technique with significant benefits useful in providing effective regional anesthesia during knee surgery.

**Summary:**

This narrative review explores the anatomy involved in knee surgery, anesthesia, clinical considerations, and potential benefits using an ultrasound guided genicular nerve block. At present, there is need for large sample size studies after ultrasound guided genicular nerve block for knee replacement surgery, evaluation of long-term outcomes with specific focus on functional recovery, rehabilitation participation, and patient centered outcomes.

## Introduction

Osteoarthritis (OA) of the knee is a major cause of chronic pain, disability, and healthcare utilization worldwide often leading patients to consider total knee arthroplasty (TKA) when conservative management fails [1]. While TKA provides substantial pain relief and return of functionality for many patients, it is associated with significant post-operative pain, with roughly 20% of patients experiencing persistent postsurgical pain three to six months postoperatively [2]. With procedural volume continuing to rise with exponentially with a rapidly aging population, optimization of perioperative analgesia becomes essential in patient’s perioperative experience and postoperative outcome.

Genicular nerve targeted therapies have become a leading topic of discussion when considering ways to minimize opioid exposure while providing effective perioperative analgesia by selectively blockading pain signal transmission from the knee joint.

Genicular nerve targeted therapy was initially utilized in radiofrequency ablation strategies for difficult-to-treat osteoarthritis pain, with blocks preformed under fluoroscopy by identifying the superomedial, superolateral, and inferomedial genicular nerve branches based on bony anatomical landmarks. Some of the major limitations of the fluoroscopic approach includes ionizing radiation exposure for the patient as well as poor visualization of soft tissue structures for the provider. However, the recent widespread adoption of ultrasound in perioperative setting offers a potential solution allowing for portable, cost effective, radiation free visualization of neural, vascular, and soft tissue structures in real time [3]. With the advent of widespread ultrasound use in perioperative anesthesia, ultrasound guided techniques for GNBs have become increasingly popular as a cornerstone of perioperative TKA multimodal analgesic management [4].

### Anatomy/Mechanism of Analgesia

The sensory innervation of the knee joint is primarily comprised of contributions from the femoral, obturator, sciatic, tibial, and common peroneal nerves [5]. The anterior capsule of the knee receives most nociceptive innervation from terminal branches referred to collectively as genicular nerves.

The genicular nerves of greatest procedural relevance are the superomedial, superolateral, and inferomedial genicular nerves [6]. The superomedial genicular nerve is derived from the terminal branches of the tibial and posterior obturator nerves, located near the adductor tubercle and medial femoral epicondyle. The superolateral genicular nerve originates from the common peroneal component of the sciatic nerve and can be found adjacent to the lateral femoral epicondyle. The inferomedial genicular nerve originates from the tibial nerve and runs along the medial tibial condyle passing beneath the medial collateral ligament [5]. Each of these genicular nerves are accompanied by genicular arteries, serving as sonographic landmarks during ultrasound guided blocks.

Historically, genicular nerve blocks have been performed using fluoroscopic imaging, relying upon osseous landmarks to locate the targeted nerve branches [7]. Under fluoroscopic imaging, the superior branches are typically targeted near the junction of the femoral shaft and epicondyles, and the inferomedial branch is targeted at the medial tibial condyle. While allowing for high quality visualization of osseous landmarks, limitations of the fluoroscopic approach include poor visualization of soft tissue like the neural and vascular structures as well as exposure to ionizing radiation for both patient and provider. Ultrasound offers the advantage of visualization of both the osseous surface, as well as the surrounding soft tissue planes without ionizing radiation exposure. Additionally, ultrasound doppler imaging has the potential to improve procedural safety with identification of the genicular arteries which run adjacent to the targeted nerve branches. Likewise, ultrasound allows for real time visualization of needle advancement and local anesthetic spread to aid in avoidance of vulnerable structures such as ligaments, tendons, and adjacent nerves. Other advantages of ultrasound over fluoroscopic imaging include the portability and relatively low cost of the imaging modality [8, 9].

The analgesic goal of the genicular nerve block is targeted at denervation of the anterior knee capsule and periarticular structures while preserving motor function [6]. The advantage of this approach is that genicular nerve blocks selectively target the distal sensory articular branches with minimal involvement of major fibers as opposed to the less selective femoral nerve and lumbar plexus block techniques which often produce significant quadriceps weakness impairing ambulation perioperatively. The inferolateral genicular nerve is typically avoided due to its anatomical proximity to the common peroneal nerve, which if blocked may cause motor blockade and postoperative foot drop. Likewise, given the limited evidence comparing local anesthetic agents and injection volumes in the context of genicular nerve blocks, it remains unclear which regimen best minimizes unintended spread while optimizing analgesia and preserving postoperative motor function to facilitate early rehabilitation. The motor sparing capability of the genicular nerve block poses potential to enhance postoperative recovery from procedures like total knee arthroplasty where preservation of quadriceps strength allows for early mobilization, reduces fall risk, and improved participation in rehabilitation exercise [10, 11]. Lastly, genicular nerve blocks offer utility as an adjunct when combined with other analgesic techniques like the adductor canal block to promote multimodal analgesic management and opioid sparing perioperative pain management.

### Fluoroscopy vs. Ultrasound Technique

Genicular nerve blocks were initially described using fluoroscopic guidance which remains a commonly utilized approach. Under fluoroscopic guidance, anteroposterior and lateral views are obtained to identify the appropriate bony targets. The needle is advanced until contact with the periosteal surface, followed by injection of contrast to confirm positioning and exclude intravascular infiltration prior to injection of local anesthetic [8, 12]. This landmark based approach has demonstrated clinical efficacy, however fluoroscopy guided genicular nerve block has several limitations including limited visualization of soft tissue and vascular structures, ionizing radiation exposure, increased procedural logistics, and the inability to visualize local anesthetic distribution in real time [7, 12, 13].

**Fig. 1:**
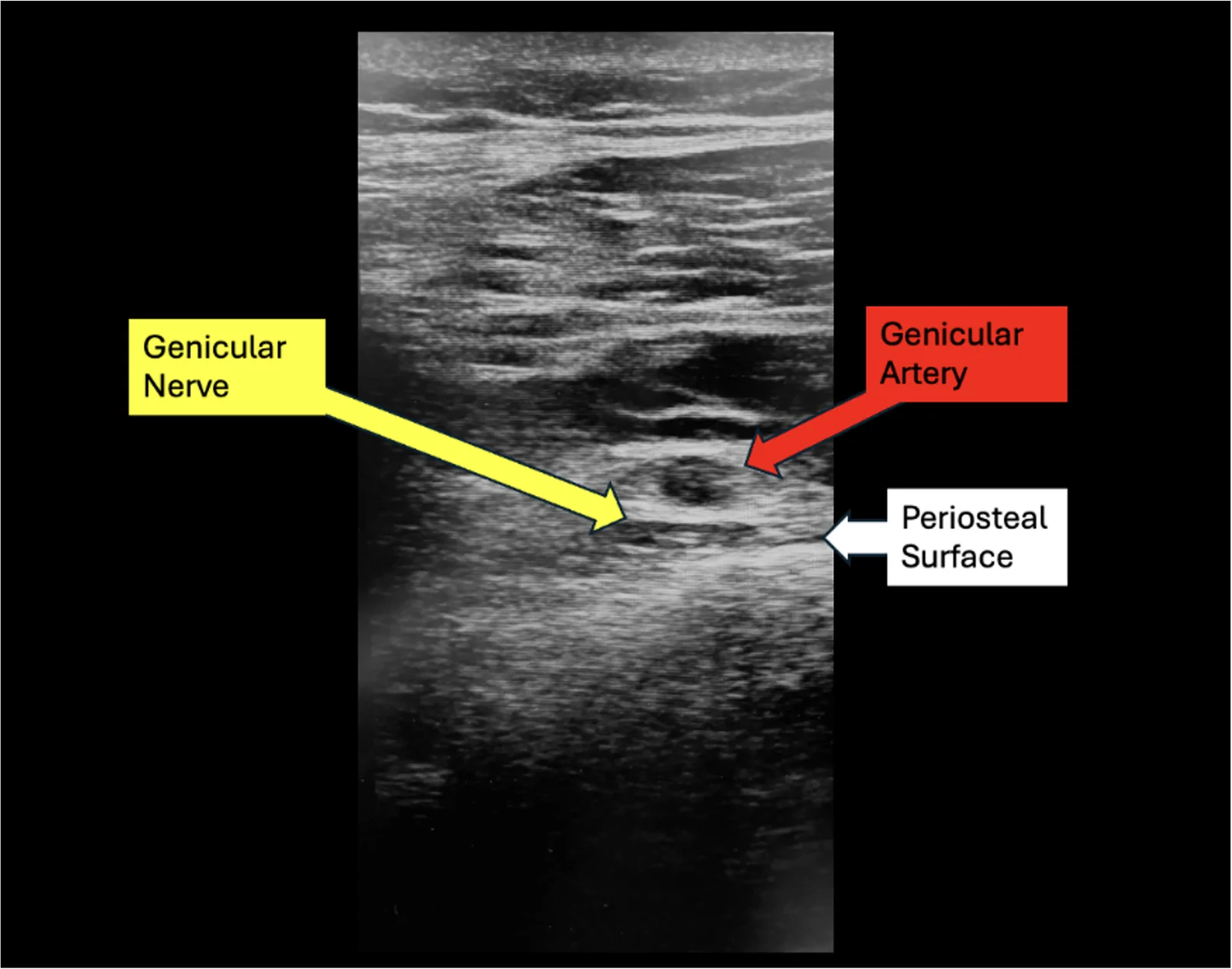
Genicular nerves are relatively easy to target with ultrasound. Highlighted are a genicular nerve, genicular artery, and the periosteal surface (original figure)

The increasing availability of ultrasound in regional anesthesia has presented ultrasound-guided genicular nerve blocks as an alternative approach providing direct visualization of soft tissue and vascular structures while still maintaining the ability to identify bony landmarks while avoiding ionizing radiation exposure for the patient and practitioner. Ultrasound guided genicular nerve blocks are typically preformed with the patient positioned supine with slight knee flexion and external rotation. The linear high frequency transducer is most appropriate given the superficial location of the targeted nerve structures. Next, the osseous landmarks are identified with the superomedial genicular nerve visualized near the medial femoral epicondyle, the superolateral genicular nerve near the lateral femoral epicondyle, and the inferomedial genicular nerve near the medial tibial condyle. Color doppler is then used to identify the corresponding arteries running adjacent to the genicular nerves, improving procedural safety. An ultrasound image highlighting genicular nerve anatomy is demonstrated in Fig. 1. The needle is then inserted utilizing an in-plane approach allowing for continuous visualization of the needle advancement as well as local anesthetic spread. Unlike fluoroscopy, the ultrasound approach allows for more precise injectate delivery with real time visualization of local anesthetic distribution without the need for a contrast test solution [8, 14, 15]. Direct visualization of the target anatomy may also allow for utilization of lower injectate volumes, potentially reducing unintended spread and preserving the motor sparing characteristics of a genicular nerve block. Additionally the portability and accessibility of ultrasound may facilitate allowing this procedure to be done in a variety of clinical settings without reliance for fluoroscopic suites [15].

### Evidence Base and Comparative Effectiveness

Genicular nerve targeted therapies are often utilized in patients with symptomatic knee osteoarthritis who are poor surgical candidates, offered as an alternative to arthroplasty. Early clinical approaches utilized genicular nerve blocks as a diagnostic tool for confirmation of efficacy prior subsequent radiofrequency ablation targeting the genicular nerve branches.

Most of this evidence was built using fluoroscopy guidance as the imaging modality, with multiple studies demonstrating improvement in pain scores and functional outcomes following genicular nerve targeted therapies for chronic osteoarthritic knee pain [9].

Much of the early literature however evaluated chronic pain populations rather than acute postoperative analgesia. With its widespread perioperative adoption, ultrasound technology became increasingly incorporated into regional anesthesia practices which was the advent of studies beginning to evaluate ultrasound guided genicular nerve block as a potential alternative to fluoroscopic guidance.

Subsequent research compared the two imaging modalities for genicular nerve blocks in patients with chronic knee osteoarthritis and found no significant difference in pain reduction, functional improvement, or treatment related outcomes between the two techniques at one and three month follow up intervals [14–16]. These findings suggest ultrasound guidance may provide comparable clinical effectiveness to fluoroscopy while offering multiple procedural advantages.

The application of ultrasound guided genicular nerve blocks has continued to gain ground in the perioperative setting, utilized as an adjunct for total knee arthroplasty analgesia. Further research evaluating ultrasound guided blockage of the superolateral, superomedial, and inferomedial genicular nerves in the setting of total knee arthroplasty (TKA) suggests that when added as part of a multimodal analgesic regimen, genicular nerve blockade results in significantly reduced opioid consumption at 24 and 28 h postoperatively [11, 17]. Additional, when comparing ultrasound guided genicular nerve blocks to surgeon administered periarticular infiltration analgesia techniques, the data suggests ultrasound guided genicular nerve blocks provided non-inferior analgesia during the first 24 h perioperatively with the added advantage of utilizing substantially lower volumes of local anesthetic [18].

Despite encouraging findings, relevant limitations remain with the current evidence base suggesting the need for large sample size studies, evaluation of long-term outcomes with specific focus on functional recovery, rehabilitation participation, and patient centered outcomes following knee surgery.

Overall, current evidence suggests that ultrasound guided genicular nerve blocks represent a promising motor sparing analgesic strategy for both chronic knee osteoarthritis and perioperative knee surgery. While fluoroscopy remains and established modality with strong evidence foundation, ultrasound guidance has demonstrated comparable analgesic efficacy with additional advantages related to safety, accessibility, and procedural efficiency.

### Clinical Applications in Anesthesiology Practice

The role of genicular nerve blocks (GNBs) in contemporary anesthesiology practice continues to evolve as perioperative pain management increasingly emphasizes opioid-sparing multimodal analgesia, preservation of motor function, and early mobilization. Traditional regional anesthetic techniques for knee surgery, including femoral nerve blocks, sciatic nerve blocks, and to a lesser extent, adductor canal blocks, may be associated with varying degrees of motor weakness that can impair postoperative ambulation [19]. In contrast, GNBs selectively target the sensory articular branches supplying the knee joint while largely sparing motor innervation. This motor-sparing characteristic makes GNB an attractive option allowing early mobilization and may facilitate shorter hospital stays [20, 21]. Given the limited research evaluating ultrasound-guided genicular nerve blocks relative to established multimodal analgesic strategies, GNB is currently best viewed as an adjunct rather than a standalone regional technique for total knee arthroplasty. Further comparative studies are needed to better define its optimal role in perioperative analgesia.

There is emerging evidence that suggests that GNB may contribute to a significant reduction in postoperative opioid requirements. In a study by Caldwell et al. evaluating patients undergoing ACL reconstruction, a circumferential genicular nerve block was associated with a mean postoperative opioid consumption of only 5.5 hydrocodone tablets (27.5 oral morphine equivalents), with 21% of patients requiring no opioid medication and no patients requiring prescription refills. When compared with historical opioid consumption following ACL reconstruction, this represented an approximate 82% reduction in opioid utilization [22]. Although further studies are needed, these findings emphasize the potential opioid-sparing benefits of genicular nerve blockade.

Potential candidates for GNB include patients undergoing TKA who would benefit from the early ambulation and sparing of motor function that GNB provides. Additionally, GNB may have a role as a significant adjunct when providing analgesia in complex revision knee arthroplasty, although further studies are required to define its efficacy in these patient populations. By reducing reliance on systemic opioids, GNB may help decrease opioid related complications such as nausea, vomiting, constipation, sedation, and respiratory depression, which will facilitate postoperative recovery and patient satisfaction.

The explosive increase in orthopedic surgery being done in the ambulatory setting with same-day discharges has created a growing need for analgesic techniques that allow for effective pain control, while reducing motor impairment and opioid requirements [23]. This creates an attractive view when looking at GNB in this setting due to the reasons mentioned earlier. Although there is a need for more research evaluating GNB in the outpatient setting, the benefits of this block suggest a promising role in outpatient TKA and other knee procedures.

### Safety, Limitations, and Future Directions

Complications associated with GNB are similar to those seen with any procedure involving needle penetration of the skin, including infection, bleeding, bruising, nerve injury, and procedural pain. The goal of any nerve block is to target a specific nerve and provide pain relief to the surgical site; however, there is always a risk of inadvertently affecting nearby structures. Unintentional injury to adjacent nerves can result in temporary or permanent numbness, weakness, or altered sensation [24].

The nerve most commonly at risk during GNB is the common peroneal nerve. This can occur when the inferolateral genicular nerve is targeted and local anesthetic spreads to the common peroneal nerve, potentially resulting in foot drop. Finally, as with any regional anesthetic technique, there is a risk of intravascular injection leading to local anesthetic systemic toxicity (LAST) or intraneural injection, which may result in nerve injury, prolonged postoperative neurologic deficits, or ischemia.

Contraindications to GNB include known allergy or hypersensitivity to local anesthetics, bleeding disorders, coagulopathies, or any state that puts a patient at higher risk for hematoma formation and at the most extreme compartment syndrome. Finally, any patient with a known active infection at the site of injection this procedure should be avoided.

When considering ultrasound versus fluoroscopy for GNB, a study by Kim et al. found that pain relief, functional improvement, and safety outcomes were similar between the two approaches. However, given the absence of radiation exposure, ultrasound guidance may offer an advantage over fluoroscopic guidance. Another study by Dagistan et al. suggested that ultrasonography may be advantageous because it allows for more precise visualization of the target location and surrounding tissues while avoiding radiation exposure. However, these advantages may be limited by factors such as operator experience with ultrasonography and variable nerve visualization among patients with different body habitus.

As this procedure becomes more widely adopted, standardization of technique will likely follow. However, although some studies have evaluated GNB, many remain inconclusive due to small sample sizes. The next step is the development of larger, sufficiently powered clinical trials comparing the role of genicular nerve block in pain management.

## Conclusion

Fluoroscopy has historically established the anatomic framework for genicular nerve block and contributed to the development of early standardized targeting techniques. Ultrasound guidance offers comparable analgesic efficacy while providing an improved safety profile, including the elimination of radiation exposure and enhanced visualization of adjacent vascular structures. In addition, ultrasound allows for more efficient integration into contemporary regional anesthesia workflows. Given these advantages, there is an anticipated shift toward broader adoption of ultrasound-guided genicular nerve blocks in perioperative pain management as experience grows and techniques become further standardized. As ultrasound-guided genicular nerve blocks become increasingly incorporated into perioperative pain management pathways, future research should focus on comparative studies identifying the optimal local anesthetic agent, concentration, injection volume, and potential adjuvants. Establishing an ideal anesthetic regimen will be essential to maximizing analgesic duration while preserving motor function to facilitate early ambulation, rehabilitation, and safe same-day discharge.

## Key References

- Fathi HM, Fahmy AM, Shaker MA, Kamel AAF. Comparison of genicular nerve block and its combination with IPACK block for analgesia and recovery after total knee arthroplasty: a randomized trial. BMC Anesthesiol. 2025 Nov 29;25(1):601. 10.1186/s12871-025-03408-0.
  - ⚬ Interesting study comparing genicular nerve block and combination with IPACK block.
- Aoyama Y, Sakura S, Nakaji Y, Yuwapattanawong K, Nikai T. The Role of Genicular Nerve Blocks in Enhancing Postoperative Pain Management After Total Knee Arthroplasty: A Retrospective Study. Anesthesiol Res Pract. 2025;2025:8827996. 10.1155/anrp/8827996 PubMed PMID: 40365112; PubMed Central PMCID: PMC12074850.
  - ⚬ Interesting study on role of genicular nerve block after total knee arthroplasty
- Yasar E, Kesikburun S, Kılıç C, Güzelküçük Ü, Yazar F, Tan AK. Accuracy of Ultrasound-Guided Genicular Nerve Block: A Cadaveric Study. Pain Physician. 2015;18(5):E899-904. PubMed PMID: 26431143.
  - ⚬ Important study on accuracy of ultrasound guided genicular nerve block using cadavers.

## Data Availability

No datasets were generated or analysed during the current study.
